# Efficacy and Safety of Low-Dose Interleukin-2 Therapy in Systemic Lupus Erythematosus: A Systematic Review

**DOI:** 10.7759/cureus.83323

**Published:** 2025-05-01

**Authors:** Murad A Akkur, Nuha A Areeshi, Ibrahim Y Haqawi, Ahmed A AlHabji, Ahmed H Altaher, Hanan A Jawkhab, Maram M Fageehi, Abdulrahman A Moafa, Ahmed I Ismael, Nouf A Al Alhadi

**Affiliations:** 1 Internal Medicine, Prince Mohammed Bin Nasser Hospital, Jazan, SAU; 2 Internal Medicine, King Fahad Military Medical Complex, Dhahran, SAU; 3 Internal Medicine, King Fahad Central Hospital, Jazan, SAU; 4 College of Medicine, Jazan University, Jazan, SAU

**Keywords:** biomarkers, immunotherapy, interleukin-2, low-dose il-2, regulatory t cells, safety, sledai, systemic lupus erythematosus

## Abstract

Systemic lupus erythematosus (SLE) is a chronic autoimmune disease characterized by immune dysregulation, including impaired regulatory T cell (Treg) function. Low-dose interleukin-2 (Ld-IL-2) therapy has emerged as a promising approach to selectively expand Tregs and restore immune tolerance in SLE. This systematic review evaluates current evidence on the efficacy and safety of Ld-IL-2 therapy in patients with SLE. A comprehensive literature search was conducted across PubMed, Scopus, Web of Science, the Cochrane Library, and the Virtual Health Library for studies published up to April 10, 2025. Eligible studies included randomized controlled trials, cohort studies, and open-label trials that investigated Ld-IL-2 therapy in adult patients with SLE. Data were extracted on study design, patient demographics, intervention details, clinical and immunologic outcomes, adverse events, and predictive biomarkers. Risk of bias was assessed using the Modified Downs and Black checklist. Seven studies met the inclusion criteria, encompassing a total of 517 patients with active SLE. All studies reported significant expansion of Treg populations following Ld-IL-2 treatment. Clinical outcomes consistently showed reductions in disease activity scores, such as SLEDAI and BILAG, with SRI-4 response rates ranging from 43% to 65.5%. Ld-IL-2 therapy was well tolerated, with adverse events primarily limited to mild injection-site reactions and flu-like symptoms. No serious treatment-related infections or concerns about immunogenicity were observed. Several studies identified baseline biomarkers, including low complement C3 levels, elevated PD-1^hi^ Tregs, and reduced CD4+ T cell counts, as predictors of treatment response. Ld-IL-2 therapy appears to be a safe and effective immunomodulatory treatment for patients with SLE, capable of enhancing Treg function and reducing disease activity. While current evidence is encouraging, larger multicenter randomized trials are warranted to establish standardized treatment protocols and validate predictive biomarkers for optimized patient selection.

## Introduction and background

Systemic lupus erythematosus (SLE) is a chronic, multisystem autoimmune disease characterized by the production of autoantibodies, immune complex deposition, and widespread organ inflammation [1]. The clinical course of SLE is highly variable, often involving flares and periods of remission. Standard treatment regimens rely on corticosteroids and immunosuppressive agents, which can lead to serious long-term complications, including infections, metabolic disturbances, and organ toxicity [2]. Despite these therapies, many patients fail to achieve sustained remission, highlighting the need for safer, more targeted immunomodulatory treatments.

One of the central immunological abnormalities in SLE is a deficiency in interleukin-2 (IL-2), a cytokine essential for the survival and function of regulatory T cells (Tregs) [3]. Tregs play a key role in suppressing autoreactive immune responses, and their impairment contributes to the pathogenesis of SLE [4]. Reduced IL-2 levels in patients with SLE impair Treg proliferation and function while permitting the expansion of pro-inflammatory effector T cells, further exacerbating autoimmune activity [5].

Low-dose IL-2 (Ld-IL-2) therapy has emerged as a promising approach to correct this immune imbalance by selectively expanding and activating Tregs without stimulating effector T cells [6]. Unlike the high-dose IL-2 used in oncology, low doses exploit IL-2’s immunoregulatory properties while avoiding systemic inflammation. Several clinical studies and early-phase trials have demonstrated that low-dose IL-2 improves Treg frequency and function, reduces disease activity scores such as SLEDAI and BILAG, and is associated with minimal adverse events [7,8].

In randomized and open-label trials, patients treated with low-dose IL-2 have shown increased CD25⁰ⁱ Treg populations, reduced anti-dsDNA titers, normalization of complement levels, and steroid-sparing effects [9,10]. These immunologic improvements translate into clinical benefits, with studies reporting higher rates of SRI-4 response and lower relapse rates than conventional treatment alone [7]. Furthermore, biomarkers such as PD-1⁰ⁱ Tregs, reduced CD4+ T cells, and elevated serum IFN-α have been explored as potential predictors of treatment response [9].

The safety profile of Ld-IL-2 has also been encouraging. Across multiple trials, treatment-related adverse effects were predominantly mild and transient, including injection-site reactions and flu-like symptoms. Importantly, no significant increases in serious infections or malignancies have been reported [3,6]. These findings position Ld-IL-2 as a viable candidate for long-term immunomodulation in SLE.

## Review

Methods

Literature Search Strategy

This review was conducted following the Preferred Reporting Items for Systematic Reviews and Meta-Analyses (PRISMA) 2020 guidelines. A systematic and exhaustive search was performed across five major electronic databases: PubMed, Scopus, Web of Science, Cochrane CENTRAL, and the Virtual Health Library, covering all publications up to April 10, 2025. The search strategy combined MeSH terms and relevant keywords such as ("Systemic Lupus Erythematosus" OR "SLE") AND ("Interleukin-2" OR "IL-2") AND ("low-dose" OR "low dose") AND ("therapy" OR "treatment"), with Boolean operators used to enhance specificity. The queries were adapted to suit the indexing systems of each database. Filters were applied to limit results to studies conducted in humans and published in English. Additionally, reference lists of included studies were manually checked to identify any potentially relevant articles not captured during the database search.

Eligibility Criteria

Inclusion and exclusion criteria were developed using the PICO framework (Population, Intervention, Comparator, Outcomes, and Study Design). Eligible studies were randomized controlled trials, prospective cohort studies, and clinical trials that met the following conditions: enrolled participants diagnosed with SLE based on recognized diagnostic criteria; investigated the use of Ld-IL-2 as a therapeutic intervention; compared IL-2 treatment to placebo, standard care, or no intervention; and reported relevant clinical or immunological outcomes, such as SLEDAI, BILAG, or SRI-4 response. Excluded studies were those published in non-English languages, non-human studies, and publications such as case reports, case series, narrative reviews, editorials, conference proceedings, or clinical trial registrations.

Study Selection

All retrieved records were independently screened by two reviewers, starting with titles and abstracts and followed by full-text assessments of potentially relevant articles. Any discrepancies or disagreements were resolved through discussion, and if needed, adjudicated by a third reviewer to reach consensus.

Data Extraction

Data extraction was carried out for all studies that met the inclusion criteria. The reviewers collected key information including study design, author and year, country, sample size, treatment duration, follow-up period, and baseline patient characteristics. Details of the intervention protocols (dose, schedule, and route of IL-2 administration) and comparator arms (if present) were also recorded. Outcomes related to clinical efficacy (e.g., SLEDAI reduction, SRI-4 response), immunologic markers (e.g., Treg frequency), and safety were systematically extracted. All data were cross-checked, and disagreements were resolved through collaborative discussion to ensure data accuracy.

Quality Appraisal

The methodological quality of the included studies was independently assessed by two reviewers using the modified Downs and Black scale for clinical trials [11]. The scale consists of 27 questions rating four categories: (i) reporting, (ii) external validity, (iii) internal validity, and (iv) power. Studies are considered of excellent quality when the final score ranges from 26 to 28, good quality if the score ranges from 20 to 25, fair quality if the score ranges from 19 to 15, and poor if the score is 14 or less. Any disagreements or discrepancies were resolved by discussion until a consensus was reached.

Results

Study Selection

The initial search identified a total of 5,238 records through electronic databases including PubMed, Scopus, Web of Science, Cochrane Library, and others. No additional records were retrieved from other sources. After removing duplicates, 3,782 unique records remained for screening. During the title and abstract screening phase, 3,741 records were excluded for not meeting the eligibility criteria.

**Figure 1:**
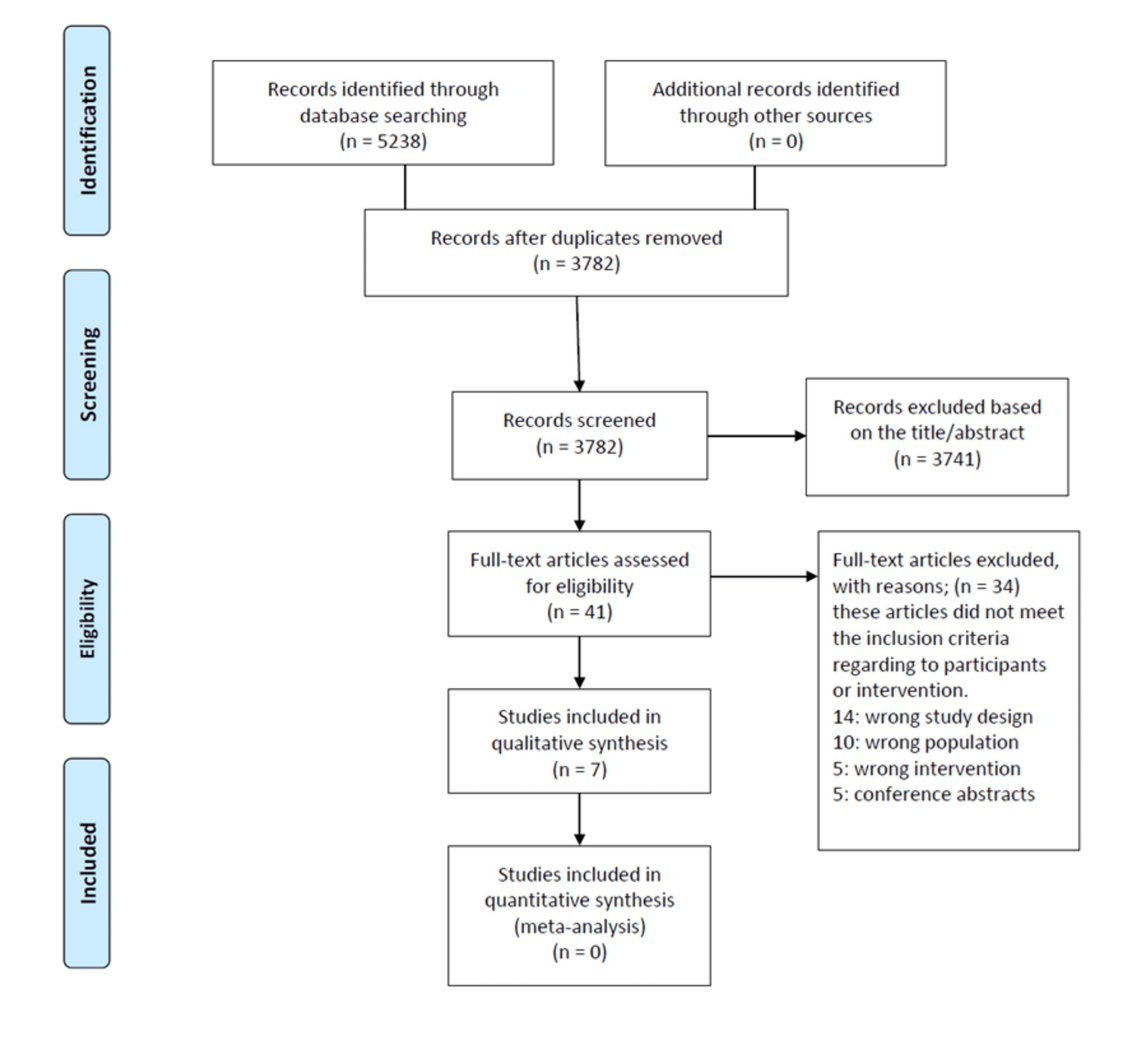
Flow diagram of the study selection process according to the Preferred Reporting Items for Systematic Reviews and Meta-Analyses (PRISMA) guidelines

A total of 41 full-text articles were then assessed for eligibility. Of these, 34 were excluded for the following reasons: 14 due to incorrect study design, 10 due to an ineligible population, 5 due to inappropriate interventions, and 5 were conference abstracts. Ultimately, seven studies [3,6-10,12] met the inclusion criteria and were included in the qualitative synthesis. No studies were eligible for quantitative synthesis (meta-analysis). This process is detailed in the PRISMA 2020 flow diagram (Figure 1).

Study Characteristics

von Spee-Mayer et al. [8] conducted a phase I/IIa open-label trial in Germany involving five adult patients with active SLE, who received 1.5 million IU/day of subcutaneous aldesleukin for five consecutive days. The study had no control group and included a one-day follow-up. Outcomes focused on Treg cell frequency, phenotype, and CD25 expression. He et al. [12] carried out a prospective open-label study in China with 40 patients aged 18-65 years (37 female patients, three male patients) who met the 1997 ACR criteria and had SELENA-SLEDAI ≥8. The intervention consisted of 1 million IU IL-2 subcutaneously every other day for two weeks per cycle, repeated for three cycles over 12 weeks. No control group was included. The study assessed SRI-4 response, disease activity reduction, modulation of Treg/TFH/TH17 subsets, and glucocorticoid tapering (Table 1).

**Table 1:** Study characteristics and outcomes of IL-2 treatment in patients with SLE This table summarizes the key characteristics and results of studies evaluating interleukin-2 (IL-2) treatment in patients with systemic lupus erythematosus (SLE). It includes information on study design, country, sample size, duration, follow-up, participant demographics, baseline SLE criteria, intervention details, control group, primary outcomes, and results. ACR: American College of Rheumatology; SLEDAI: Systemic Lupus Erythematosus Disease Activity Index; SRI-4: Systemic Lupus Erythematosus Responder Index 4; Treg: Regulatory T cells; TFH: Follicular helper T cells; TH17; T helper 17 cells; BILAG: British Isles Lupus Assessment Group index; PGA: Physician's Global Assessment; CLA⁺: Cutaneous lymphocyte antigen-positive. Reference numbers are provided for each study.

Author(s), Year	Study Design	Country	Sample Size	Age/Gender	SLE Criteria & Baseline	Treatment Duration/Follow-up	Intervention	Control	Outcomes	Results
Rosenzwajg et al., 2018 [6]	Open-label phase I/IIa trial	France	46 patients	23–75 years; mixed gender	Mild to moderate SLE; on stable therapy ≥2 months	6-month treatment + 2-month follow-up	1 million IU/day IL-2 × 5 days, then q2w for six months	None	Treg expansion, inflammatory markers, clinical scores	Treg expansion, improved inflammation profile, safety confirmed
He et al., 2020 [7]	RCT, double-blind, placebo-controlled	China	60 patients	18–65 years; mixed gender	ACR criteria; active disease, SLEDAI ≥4	12-week treatment + 12-week follow-up	1 million IU IL-2 SC every other day × 2 weeks/cycle × 3 cycles	Placebo	SRI-4, SLEDAI, proteinuria, CD25^hi Tregs, safety	SRI-4 at week 24: 65.5% vs. 36.7% (placebo); p = 0.027; no serious infections
von Spee-Mayer et al., 2016 [8]	Phase I/IIa open-label trial	Germany	5 patients	Not fully detailed; adults	ACR; active disease, SLEDAI scores referenced	5-day treatment + 1-day follow-up	1.5 million IU/day IL-2 (aldesleukin) SC × 5 days	None	Treg cell frequency & phenotype, CD25 expression	Treg expansion, improved CD25 expression, linked to IL-2 deficiency
Raeber et al., 2024 [9]	Single-arm Phase II trial	Switzerland	12 female patients	Adult female patients	Active disease; SLEDAI, BILAG, serological markers	9 weeks total, 4 cycles	1.5 million IU/day IL-2 × 5 days/cycle × 4 cycles	None	Treg expansion, skin/blood phenotyping, cytokines	Treg subsets expanded; CLA⁺ skin-homing Tregs ↑; clinical response noted
Feng et al., 2024 [10]	Retrospective cohort study	China	342 patients	Mean 39 years; 91% female	1997 ACR; active disease, SLEDAI ≥4	≥3 months treatment, variable follow-up	1 million IU IL-2 SC every other day × 2 weeks, 2-week break/cycle	None	SRI-4, LLDAS, CD4/CD8/NK/Treg markers, clinical labs	SRI-4 response: 65.5%; predictors: low C3, rash, renal involvement
He et al., 2016 [12]	Prospective open-label study	China	40 patients	18–65 years; 37 F, 3 M	1997 ACR; SELENA–SLEDAI ≥8	12 weeks, 3 IL-2 cycles	1 million IU IL-2 SC every other day × 2 weeks/cycle × 3 cycles	None	SRI-4, SELENA–SLEDAI, Treg/TFH/TH17 modulation	SRI-4: 89.5%; Tregs ↑, TFH & TH17 ↓; SLEDAI ↓; steroids reduced in 91.9%
Humrich et al., 2019 [13]	Phase I/IIa uncontrolled trial	Germany	12 patients	18–75 years; refractory SLE	ACR; moderate-to-severe despite ≥2 therapies	62-day treatment + follow-up	1.5 million IU/day aldesleukin SC × 5 days/cycle × 4 cycles	None	Treg % increase, SLEDAI, BILAG, PGA	Tregs ↑ significantly; SLEDAI ↓; no serious AEs; well tolerated

Rosenzwajg et al. [6] conducted an open-label phase I/IIa trial (TRANSREG) in France involving 46 patients aged 23-75 years with mild to moderate SLE on stable therapy for at least two months. Treatment included 1 million IU/day of IL-2 for five days followed by maintenance injections every two weeks for six months, with a two-month follow-up. The study had no control group and assessed Treg expansion, inflammatory markers, and clinical scores. Raeber et al. [9] conducted a single-arm phase II trial in Switzerland with 12 adult female SLE patients receiving 1.5 million IU/day aldesleukin for five days per cycle over four cycles spanning nine weeks. Outcomes included Treg expansion, immune phenotyping of skin and blood, and cytokine profiling.

Feng et al. [10] presented a retrospective cohort study in China involving 342 patients (91% female, mean age 39 years) with SLEDAI ≥4. Patients received 1 million IU IL-2 subcutaneously every other day for two weeks, followed by a two-week break per cycle, over at least three months. No control group was used. Outcomes included SRI-4, LLDAS, immune subset analysis (CD4, CD8, NK, Treg), and clinical labs. He et al. [7] conducted a randomized, double-blind, placebo-controlled trial in China involving 60 patients aged 18-65 years with active SLE (SLEDAI ≥4). Patients received 1 million IU IL-2 every other day for two weeks per cycle across three cycles, with placebo used for comparison. Outcomes included SRI-4, changes in disease activity, proteinuria, CD25^hi Treg frequency, and safety over a 24-week follow-up.

Humrich et al. [13] performed a phase I/IIa uncontrolled trial in Germany involving 12 patients aged 18-75 years with refractory SLE. The intervention consisted of 1.5 million IU/day aldesleukin for five days per cycle across four cycles, with 62 days of treatment plus follow-up. No control group was included. The study evaluated Treg percentage increases and changes in SLEDAI, BILAG, and PGA scores.

Quality Assessment

The study by von Spee-Mayer et al. [8] scored a total of 20 out of 27 on the quality assessment checklist. It achieved 8 points for reporting, 2 for external validity, 5 for internal validity related to bias, 4 for confounding, and 1 for power. He et al. [7] scored 26 overall, with 10 points in reporting, 2 in external validity, 7 in internal validity (bias), 6 in internal validity (confounding), and 1 point for power. Rosenzwajg et al. [6] had a total score of 22, including 9 for reporting, 2 for external validity, 5 for bias, 5 for confounding, and 1 for power. Raeber et al. [9] received a total of 23 points, comprising 9 for reporting, 2 for external validity, 6 for bias, 5 for confounding, and 1 for power. Feng et al. [10] also achieved 23, with 9 points in reporting, 2 for external validity, 6 for bias, 5 for confounding, and 1 for power. He et al. [12] scored 24, with full marks in reporting, and strong scores across bias and confounding. Finally, Humrich et al. [13] scored 23, consisting of 9 for reporting, 2 for external validity, 6 for bias, 5 for confounding, and 1 for power (Table 2).

**Table 2:** Methodological quality assessment of included studies using the modified Downs and Black scale The table presents the scores for each methodological quality category, including reporting, external validity, internal validity (bias), internal validity (confounding), and power, as assessed using the modified Downs and Black scale [11]. The total score for each study is also provided, with reference numbers in the last column.

Study	Reporting (10)	External Validity (3)	Internal Validity – Bias (7)	Internal Validity – Confounding (6)	Power (1)	Total (max 27)
Rosenzwajg et al. [6]	9	2	5	5	1	22
He et al. [7]	10	2	7	6	1	26
von Spee-Mayer et al. [8]	8	2	5	4	1	20
Raeber et al. [9]	9	2	6	5	1	23
Feng et al. [10]	9	2	6	5	1	23
He et al. [12]	10	2	6	5	1	24
Humrich et al. [13]	9	2	6	5	1	23

Effect of Interventions

Clinical efficacy across studies: In the study by Feng et al. [10], 43.3% of 314 patients achieved the SRI-4 response at 12 weeks, with a reduction in SLEDAI from a median of 11 to 4. He et al. [7] reported an SRI-4 response of 65.5% at week 24 in the IL-2 group compared to 36.7% in the placebo group. von Spee-Mayer et al. [8] showed that 8 out of 12 patients were clinical responders, with a reduction in SLEDAI from 10 to 5 by day 62. Raeber et al. [9] reported reductions in SELENA-SLEDAI, BILAG, and PGA scores. In Humrich et al. [13], 20 out of 40 patients responded to treatment based on SRI-4 criteria. In the study by Rosenzwajg et al. [6], clinical global impression activity and severity scores improved at months 3 and 6.

Complete renal remission in lupus nephritis was reported in 53.8% of patients in the study by He et al. [7] compared to 16.7% in the placebo group. In the study by von Spee-Mayer et al. [8], clinical manifestations such as arthritis, rash, and alopecia resolved in most patients. In the study by Feng et al. [10], responders showed improvements in rash, leukopenia, and thrombocytopenia. In the study by Raeber et al. [9], anti-dsDNA decreased and serum C3c increased.

In the study by He et al. [7], 44.8% of patients had a ≥50% prednisone dose reduction. In the study by Raeber et al. [9], prednisone use was reduced. In the study by Humrich et al. [13], corticosteroids were tapered according to EULAR guidelines.

Immunological response and mechanisms: In the study by Rosenzwajg et al. [6], the mean Treg percentage increased from 5.8% to 11.1% by day 8, with persistence over six months. In the study by von Spee-Mayer et al. [8], 11 out of 12 patients had ≥2-fold increases in CD25^hi Tregs by day 62. In the study by He et al. [7], significant expansion of Tregs was confirmed by flow cytometry. In the study by Humrich et al. [13], Tregs increased over 12 weeks. In the study by Raeber et al. [9], Tregs with FOXP3^+, CD25^+, and CD127^lo phenotypes expanded significantly.

Raeber et al. [9] showed increased frequencies of CLA^+ skin-homing Tregs and CD38^+HLA-DR^+ DP Tregs. He et al. [12] reported in vitro conversion of CD25^neg and CD25^int to CD25^hi Tregs and increased Bcl-2 and Foxp3 expression. Functional Treg assays in Humrich et al. [13] and Raeber et al. [9] showed preserved suppressive capacity. In the study by Rosenzwajg et al. [6], the Treg:Teff ratio increased by 2.17-fold. In the study by He et al. [12], Tcons did not expand after IL-2. In the study by Raeber et al. [9], subsets such as Tfr, Treg1, and Treg2 expanded, while conventional Th subsets remained stable.

Biomarkers and immunologic correlates: Feng et al. [10] identified lower baseline CD4+, CD8+ T cells, and NK cells and higher B cells as predictors of SRI-4 response. Multivariate predictors included rash, proteinuria, occult blood, urine casts, low C3, and low CD4+ T cells, with a prediction AUC of 0.933. Raeber et al. [9] associated SLEDAI improvements with low RTE Tregs, high PD1^hi Tregs, low CD122^+ CD8+ Tcm, and high IFNα.

In the study by He et al. [7], anti-dsDNA levels decreased and serum C3 and C4 increased. In the study by Feng et al. [10], C3, C4 increased and IgG and anti-dsDNA decreased. von Spee-Mayer et al. [8] showed reduced anti-dsDNA and increased complement. Raeber et al. [9] confirmed similar changes. Humrich et al. [13] also reported decreases in IgG and increases in C3 and C4.

Safety and tolerability: He et al. [7] reported no serious adverse events; injection site reactions occurred in 31.0% of IL-2 patients. Infection rates were 6.9% in the IL-2 group versus 20.0% in the placebo group. Raeber et al. [9] reported mild flu-like symptoms and injection site reactions. Feng et al. [10] noted injection site reactions in 6.7%, transient fever in 1.5%, and minor infections. Rosenzwajg et al. [6] observed no serious adverse events and reported injection site reactions as the most common mild adverse event. No anti-IL-2 antibodies were detected in the study by Raeber et al. [9] or Rosenzwajg et al. [6]. Humrich et al. [13] reported no serious adverse events.

Discussion

The systematic review evaluated the efficacy and safety of Ld-IL-2 therapy in patients with SLE. The findings from the included studies are consistent with existing literature, highlighting the potential of Ld-IL-2 in modulating immune responses and improving clinical outcomes in SLE.

Efficacy of Ld-IL-2 in SLE

Multiple studies within the review demonstrated that Ld-IL-2 therapy leads to a significant expansion of regulatory T cells (Tregs), which are crucial for maintaining immune tolerance. For instance, von Spee-Mayer et al. reported a ≥2-fold increase in CD25^hi FOXP3^+ Tregs in 92% of patients by day 62 [8]. Similarly, He et al. observed that Ld-IL-2 restored CD25 expression in Tregs within 24 hours, enhancing their survival and function [12]. Raeber et al. further demonstrated that Ld-IL-2 induced marked expansion of both circulating and skin-homing Treg subsets, confirmed by phenotypic and functional analyses [9]. This is consistent with evidence from the study by Humrich et al., who showed that Ld-IL-2 therapy significantly increased CD4^+ CD25^hi CD127^lo Tregs and improved their suppressive capacity in refractory SLE patients [13]. Likewise, Su et al. in a systematic review and meta-analysis confirmed the robust and selective expansion of Tregs in autoimmune rheumatic diseases treated with Ld-IL-2 [14]. Collectively, these findings align with previous research indicating that Ld-IL-2 selectively expands Tregs without activating effector T cells, thereby restoring immune balance in autoimmune conditions like SLE [5].

Supporting Evidence From Other Investigations

Several independent investigations outside the core studies included in the current review also support the clinical efficacy of Ld-IL-2 therapy in SLE. Saadoun et al. conducted one of the earliest open-label trials on Ld-IL-2 in SLE, demonstrating significant reductions in disease activity scores and improvements in complement levels after a 5-day IL-2 course [15]. In a subsequent follow-up study by Klatzmann and Abbas, Ld-IL-2 was noted to induce durable clinical responses in SLE patients, especially when used early in the disease course [5]. Additionally, Todd et al. investigated the immunoregulatory effects of IL-2 in autoimmune diseases, reporting improved serologic markers and organ-specific symptoms in SLE patients after repeated Ld-IL-2 cycles [16]. More recently, He et al. highlighted the steroid-sparing effects of IL-2 in SLE, with a significant proportion of patients achieving disease remission on reduced corticosteroid doses [17]. These findings, derived from independent clinical cohorts, reinforce the notion that Ld-IL-2 is not only immunologically active but also clinically beneficial across diverse SLE populations.

Safety and Tolerability of Ld-IL-2

The tolerability of Ld-IL-2 therapy has consistently been supported by external investigations. Saadoun et al. reported that in patients with autoimmune vasculitis, Ld-IL-2 was well-tolerated, with adverse events limited to mild injection-site reactions and transient flu-like symptoms [15]. In a separate dose-escalation study, Todd et al. observed no dose-limiting toxicities among autoimmune patients receiving Ld-IL-2, further supporting its safety in immunomodulatory contexts [16]. Importantly, Koreth et al. found that Ld-IL-2 was not associated with opportunistic infections or autoimmunity flare-ups in a cohort of patients with chronic graft-versus-host disease, a population particularly sensitive to immune activation [18]. Collectively, these studies suggest that Ld-IL-2 maintains a favorable safety profile, making it a viable option for long-term immunoregulatory therapy.

Predictive Biomarkers for Response to Ld-IL-2 Therapy

Recent literature has emphasized the utility of immunological biomarkers in predicting response to Ld-IL-2 therapy. For example, Rosenzwajg et al. noted that patients with lower baseline frequencies of effector T cells and higher levels of activated Tregs demonstrated greater clinical improvement after IL-2 treatment [6]. Moreover, Long et al. identified that elevated serum levels of soluble CD25 and decreased PD-1^hi expression on Tregs at baseline were associated with more robust immunologic and clinical responses [19]. Additionally, Castela et al. suggested that patients with type I interferon signatures, which are common in SLE, may have differential responses to IL-2 due to altered IL-2 receptor expression [20]. These findings underscore the growing relevance of personalized therapy guided by baseline immune signatures, which may help stratify patients for optimal benefit from Ld-IL-2.

Limitations and Future Directions

While this review provides compelling evidence for the efficacy and safety of Ld-IL-2 in SLE, several limitations should be noted. These include the heterogeneity of study designs and the small sample sizes in some trials. Large-scale, multicenter randomized controlled trials are needed to confirm these findings and to establish standardized protocols for Ld-IL-2 administration in SLE. Additionally, further research into predictive biomarkers may enhance patient selection and optimize treatment outcomes.

## Conclusions

In summary, the systematic review highlights the promising therapeutic potential of Ld-IL-2 in the treatment of SLE. The evidence consistently demonstrates that Ld-IL-2 induces selective expansion of Tregs, which play a crucial role in immune tolerance and regulation. This expansion leads to a reduction in disease activity, as indicated by improvements in clinical scores and serologic markers across multiple studies. Furthermore, Ld-IL-2 therapy is associated with a favorable safety profile, with most adverse events being mild and transient, such as injection-site reactions and flu-like symptoms.
